# Exploring the relationship between Alzheimer's disease and lower extremity isokinetic muscle strength in community-dwelling older adults: A cross-sectional observational study

**DOI:** 10.1177/13872877261471888

**Published:** 2026-07-29

**Authors:** Marcos Paulo Braz De Oliveira, Silvia Lanziotti Azevedo Da Silva, Natália Oiring De Castro Cezar, Carla Rigo Lima-Truax, Brendon David Truax, Sónia Brito-Costa, Anielle Cristhine De Medeiros Takahashi, Paula Regina Mendes Da Silva Serrão, Larissa Pires De Andrade

**Affiliations:** 1 Physical Therapy Department, Federal University of São Carlos, São Carlos, São Paulo, Brazil; 2 Collective Health Department, 28113 Federal University of Juiz de Fora, Juiz de Fora, Brazil; 3 Medicine Department, State University of Minas Gerais, Passos, Brazil; 4 Physical Therapy Department, 9968 The University of Alabama at Birmingham, Birmingham, AL, USA; 5 Medicine Department, 9968 The University of Alabama at Birmingham, Birmingham, AL, USA; 6 Centre for Research and Innovation in Education (inED), Polytechnic University of Coimbra, Coimbra, Portugal; 7 Polytechnic University of Coimbra, Institute for Applied Research, Coimbra, Portugal

**Keywords:** aged, Alzheimer's disease, dementia, isokinetic dynamometry, muscle strength

## Abstract

**Background:**

Older adults diagnosed with Alzheimer's disease (AD) often experience diminished muscle strength, highlighting the continued importance of evaluating muscle function in this population.

**Objective:**

To examine the relationship between AD and isokinetic lower limb muscle strength in older adults.

**Methods:**

A cross-sectional observational study was conducted to examine the association between AD and lower limb isokinetic muscle strength in community-dwelling older adults. AD diagnosis was the independent variable; dependent variables included peak torque, average peak torque, and total work. A standardized isokinetic protocol adapted for older adults with cognitive impairment was used to assess knee (flexion/extension at 60°/s and 180°/s) and ankle (dorsiflexion/plantar flexion at 30°/s) strength. Crude and adjusted linear regression models evaluated statistical associations, with significance set at *p* ≤ 0.05.

**Results:**

A total of 80 older adults were included, 50% of whom had a diagnosis of AD. The crude model's analysis showed an association between AD and all knee and ankle isokinetic measurements. When the models were adjusted, the association was maintained only for ankle peak torque (β1 = -16.23; *p* *<* 0.048) and ankle average peak torque (β1 = -13.65; *p* *<* 0.049) at 30°/s (plantar flexion).

**Conclusions:**

Alzheimer's disease is associated with lower ankle plantar flexors isokinetic muscle strength in community-dwelling older adults. No significant associations between AD and knee (flexors and extensors) and ankle (dorsiflexors) isokinetic muscle strength were found.

## Introduction

Alzheimer's disease (AD) is marked by progressive cognitive deterioration, particularly affecting memory, and may also present with behavioral symptoms such as neuropsychiatric disorders.^1^ Beyond cognitive impairment, individuals with AD may exhibit functional limitations, including compromised postural stability,^2^ altered gait patterns,^3^ and diminished muscular strength.^4^ Different muscle groups of the lower limbs play distinct roles in functionality and fall risk, with muscles acting on the knee and ankle joints being more closely related to functional activity performance and postural control, respectively.^5, 6^ Despite these observations, the relationship between AD and lower limb muscle strength remains underexplored and warrants further investigation in this population.

In older adults with AD, lower extremity muscle strength is assessed through functional clinical tests, such as the 5-times and 30-s (30STS) sit-to-stand tests.^7^ Most of these tests do not consider the limitations associated with this population, such as comprehension challenges,^8^ which can interfere with the results by either underestimating or overestimating lower extremity muscle strength. However, Oliveira et al. (2022) showed that an adapted and standardized protocol with the isokinetic dynamometer is a reliable muscle strength assessment for this population.^9^

The isokinetic dynamometer is used to evaluate joint torque therefore, inferring muscle strength for the targeted joint.^10, 11^ When compared to commonly used functional clinical tests, the isokinetic evaluation provides more sensitive and accurate measurements of muscle strength such as peak torque (maximum muscle strength), average peak torque (the average maximum muscle strength) and total work (ability of the muscle group to maintain maximum muscle strength).^10, 11^ In addition, the isokinetic dynamometer allows the evaluation of concentric muscle strength (i.e., the total length of the muscle shortens as tension is produced) and isometric muscle strength (i.e., the length of the muscle remains relatively constant as tension is produced).^12^ According to the literature, a reduction in concentric and isometric muscle strength with increasing age is observed with a greater reduction happening in the lower extremity when compared to the upper extremity.^13^

The reduction in lower extremity muscle strength is one of the contributing factors for the functional decline observed in older adults with AD,^14^ and its evaluation by a gold standard test, such as the isokinetic dynamometer,^11^ is essential to define the association between the presence of AD and reduced muscle strength. This precise and accurate assessment can better guide researchers and clinicians working in the rehabilitation and functional maintenance of older adults. In this context, this study aims to investigate the association between AD and lower extremity isokinetic muscle strength in community-dwelling older adults. It has been hypothesized that community-dwelling older adults with AD present reduced lower extremity isokinetic muscle strength when compared to without-AD older adults.

## Methods

### Study design

This cross-sectional observational study was conducted at the Multidisciplinary Research in Older Adults’ Health and Isokinetic Dynamometry Laboratories within the Department of Physical Therapy at the Federal University of São Carlos (UFSCar), São Carlos, São Paulo, Brazil. Ethical approval was granted by the UFSCar Research Ethics Committee (CAAE: 88921118.4.0000.5504) with written informed consents obtained from all participants or their legal representatives. Data collection spanned from February to December 2019. The study adhered to the Strengthening the Reporting of Observational Studies in Epidemiology (STROBE) guidelines.^15, 16^

### Eligibility criteria

Eligibility criteria for participation included: (1) being 65 years of age or older, (2) community-dwelling, (3) both sexes, and (4) having no diagnosed musculoskeletal conditions affecting the knee or ankle. Recruitment was conducted through the School Health Unit at UFSCar, as well as through established community initiatives such as the Open University for Older Adults and local family health programs in São Carlos, São Paulo, Brazil.

### Independent variable

The primary independent variable in this study was the presence of a clinical diagnosis of AD, established according to criteria outlined in the Diagnostic and Statistical Manual of Mental Disorders, Fifth Edition (DSM-V).^17^ Participants were categorized into mild or moderate stages of AD using the Clinical Dementia Rating (CDR) scale,^18, 19^ which evaluates dementia severity across six domains: memory, orientation, judgment/problem-solving, community relationships, home and hobbies, and personal are. The CDR assigns scores reflecting dementia progression: 0.5 (very mild), 1 (mild), 2 (moderate), and 3 (severe).^18, 19^ Classification was performed by experienced researchers trained in the use of the CDR, as documented in previous research.^20–22^

Based on the study's independent variable, participants were categorized into two groups: those with AD and those without. Inclusion criteria for the AD group required a confirmed medical diagnosis of AD, classification within the mild or moderate stages of the disease, and clinical stability at the time of assessment. Exclusion criteria included inability to follow basic instructions, diagnosis of dementia from non-AD etiologies (e.g., Lewy body, vascular, frontotemporal), presence of other neurodegenerative conditions (e.g., Parkinson's disease) or non-degenerative neurological disorders (e.g., stroke), and a current diagnosis of depression.

For the non-AD group, eligibility required preserved cognitive function, demonstrated by a score of 78 or higher on the Addenbrooke's Cognitive Examination-Revised (ACE-R),^23, 24^ and absence of diagnostic criteria for mild cognitive impairment or dementia.^17, 25^ Individuals with neurological disorders or a diagnosis of depression were excluded from this group.

### Dependent variables

The dependent variables of the study included isokinetic measurements such as peak torque, average peak torque, and total work, and were assessed in all older adults. The isokinetic evaluation protocol selected for older adults with AD was published in a previous study.^9^ This protocol was adapted and standardized for these individuals and the study showed that isokinetic dynamometry is a reliable assessment tool in this population.^9^ In the present study, the assessments were performed using a Biodex Multi-Joint System 3 isokinetic dynamometer (Biodex Medical Inc., Shirley, NY, USA), with a sampling frequency of 100 Hz. Knee muscle strength was measured in extension and flexion, with angular velocities of 60°/s (five maximum voluntary repetitions) and 180°/s (15 maximum voluntary repetitions), with a total amplitude of 70°, starting from the 90° knee flexion position (0° = full extension); and in concentric and isometric modes. Ankle muscle strength was measured in plantar flexion and dorsiflexion, with angular velocity of 30°/s (five maximum voluntary repetitions), with a total amplitude of 45°, from the position of 35° of plantar flexion to 10° of ankle dorsiflexion (0° = neutral position), and in concentric mode. Both groups (i.e., older adults with- and without-AD) performed the same isokinetic assessment protocol.

### Demographic and clinical control variables

Variables selected to characterize the older adults and to control the models included demographic aspects such as age, sex, and body mass index (BMI), and clinical variables such as muscle strength, level of physical activity, cognitive function, and depressive symptoms. Six assessment tools were used to determine the clinical control variables: 1) handgrip strength using a Jamar^®^ manual dynamometer (Model PC-5030J1, Fred. Sammons, Inc., Burr Ridge, IL, USA). The older adults were instructed to perform the greatest force by maintaining the contraction for six seconds. Three measurements were performed on the dominant hand and averaged to a single score (i.e., higher mean scores represent better performance).^26^ Lower extremity muscle strength via 2) the 30STS, and 3) the calf-rise senior test (CRST). In the 30STS, older adults were instructed to get up and sit down from the chair for 30 s as quickly as possible.^27^ In the CRST test, the older adults were instructed to raise and lower their heels bilaterally for 30 s as quickly as possible.^28, 29^ Physical activity levels were evaluated using the 4) Modified Baecke Questionnaire for Older Adults (MBQOA), a tool comprising 10 items that assess engagement in daily routines, leisure activities, and sports participation. Lower scores on the MBQOA indicate reduced physical activity.^30, 31^ For participants diagnosed with AD, the questionnaire was administered through their designated caregiver to ensure reliable reporting. The criteria to be considered as a designated caregiver included caregivers, family members or guardians who stayed with the older adult for a minimum of half a day for at least four times a week. Cognitive function was assessed by 5) ACE-R). The ACE-R evaluates the domains of attention and orientation, memory, fluency, language and visuospatial skills, with a maximum score of 100 points (i.e., higher scores represent better performance).^23, 24^ Depressive symptoms were assessed using the 6) Geriatric Depression Scale (GDS), which consists of 15 affirmative and negative questions where ‘yes’ represents the presence of a symptom, and ‘no’ the absence of symptom (i.e., scores > 5 represent the presence of depressive symptoms).^32, 33^

### Study procedures

Study assessments were divided across four days. An anamnesis form was developed for the assessments and is presented in Supplemental Material 1. On day 1, the older adults were recruited. On day 2, the eligibility criteria were applied and older adults were classified as with- or without-AD based on the inclusion/exclusion criteria. On day 3, the older adults included in the study underwent demographic and clinical assessments. On the same day, the MBQOA was administered to the caregiver.^30, 31^ On day 4, older adults underwent the isokinetic assessment protocol. Isokinetic assessments were performed by a single evaluator, a physical therapist with three years of experience in this type of evaluation. Every effort was made to keep the assessments consistent on days 3 and 4 with the same time of the day (morning or afternoon) and the same team members being selected.

### Statistical analysis

Descriptive variables were shown as mean ± standard deviation for continuous variables, and as relative and absolute frequencies for categorical variables. Continuous control variables were assessed for normality using the Shapiro-Wilk test. To determine the control variables, normally distributed data were compared between the with- and without-AD groups using the independent-samples t-test, and those with a non-normal distribution were compared using the Mann-Whitney test. Categorical variables were compared for the same purpose using the chi-squared test. Variables found to be significant (*p* *<* 0.05) in the previous analyses were included in the adjusted linear regression models. The association between AD and isokinetic muscle strength was initially evaluated by crude linear regression models for each dependent measure of isokinetic muscle strength, with the AD diagnosis being the independent variable. Then, for the measures that were associated with the AD diagnosis in the crude model, models adjusted for demographic and clinical variables were constructed with *p* *<* 0.05 in the univariate analysis. The associations were measured in Beta (β) and *p values,* and *p* < 0.05 values were considered significant. The explanatory power of the model was evaluated by the adjusted R^2^. The assumptions of the models were evaluated, and all models were considered ideal by the criteria of normality, independence of residuals and homoscedasticity. Multicollinearity among the independent variables was assessed using the Variance Inflation Factor (VIF), and all values were below 10, indicating the absence or low level of multicollinearity and adequate stability of the coefficients. The analyses were performed using the statistical software R (version 4.1.0; https://www.R-project.org), considering a significance level of *p* < 0.05.

## Results

A total of 80 older adults were included in the study, 50% of whom had an AD diagnosis. All older adults underwent the demographic and clinical assessments (day 3) and the isokinetic assessment protocol (day 4). The older adults performed the isokinetic assessments correctly and with adequate range of motion. Therefore, it can be said that the older adults understood the adapted and standardized instructions of the isokinetic assessment protocol. Follow-up calls were conducted between 24 and 72 h after isokinetic assessments and no symptoms of fatigue or muscle soreness were reported. The flowchart of the older adults in the study is shown in Figure 1.

**Figure 1.**
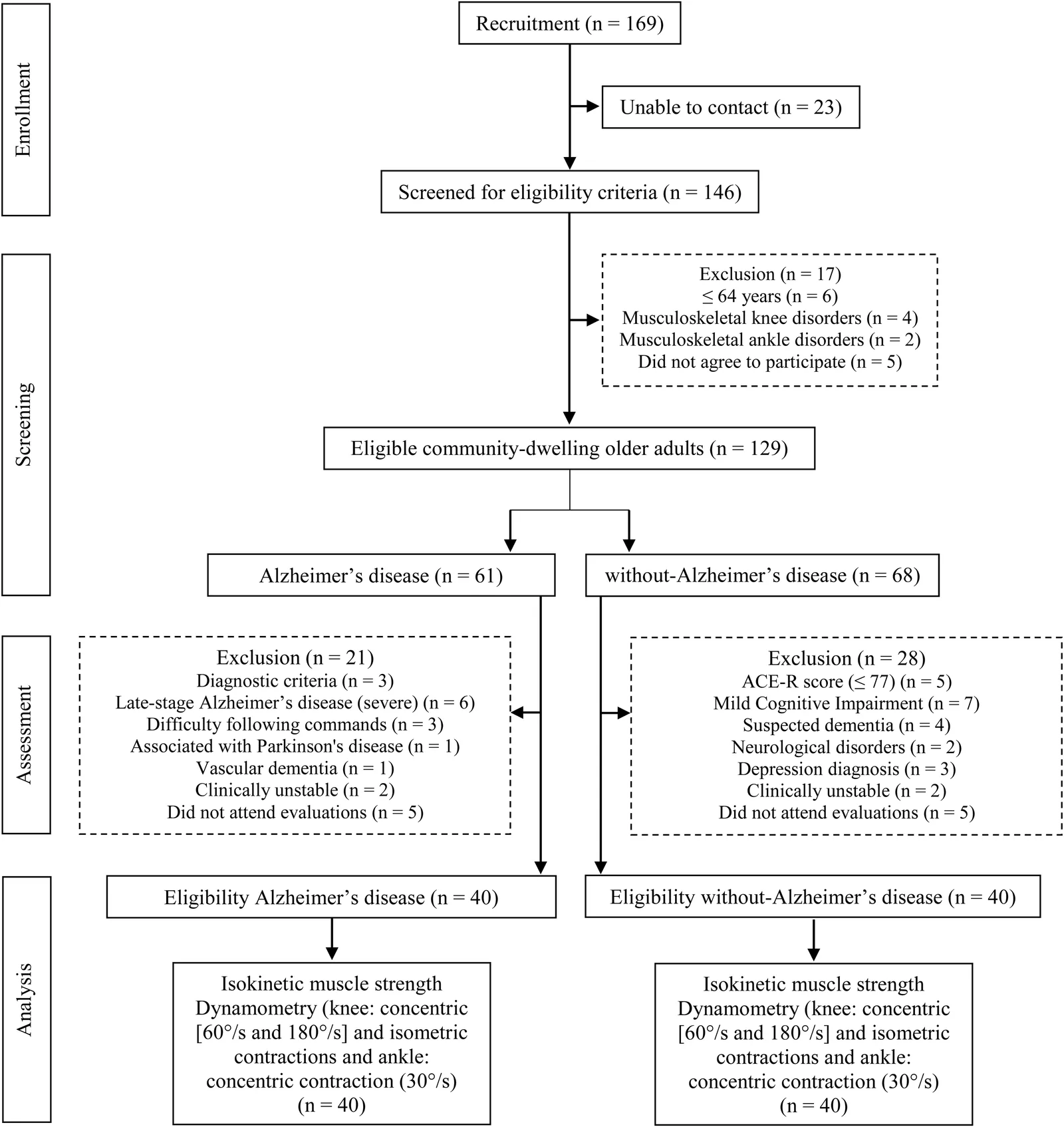
Flow diagram of older adults. ACE-R: Addenbrooke's Cognitive Examination – Revised.

The older adults with- and without-AD showed similarities in relation to sex (*p* = 0.064) and depressive symptoms (*p* = 0.082). Significant differences for the other demographic and clinical control variables were found between the groups. The with-AD group presented higher mean age (*p* = 0.007) and lower BMI (*p* = 0.046) compared to the without-AD group. The with-AD group also had lower mean muscle strength (handgrip strength [*p* < 0.004], 30STS [*p* < 0.001] and CRST [*p* < 0.001]), lower level of physical activity (MBQOA [*p* = 0.001]) and worse cognitive function ACE-R [*p* < 0.001]) compared to the without-AD group. The total and group-divided sample characteristics are shown in Table 1.

**Table 1.** Characteristics of the older adults.

Variables	All older adults (n = 80)	AD (n = 40)	Without-AD (n = 40)	*p*
Age, years, $\bar{X}$ (SD) (range)	76.70 (5.84) (66–95)	78.42 (5.95) (66–89)	74.97 (5.27) (66–95)	0.007^a^*
Sex				
Female, n (%)	51 (63.75%)	27 (67.50%)	24 (60.00%)	0.641^c^
Male, n (%)	29 (36.25%)	13 (32.50%)	16 (40.00%)	
BMI, kg*/*m^2^, $\bar{X}$ (SD)	26.95 (3.96)	26.07 (3.46)	27.83 (4.27)	0.046^a^*
Handgrip strength, kgf, $\bar{X}$ (SD)	24.00 (8.34)	21.49 (7.81)	26.51 (8.19)	0.004^b^*
30STS, rep, $\bar{X}$ (SD)	7.67 (2.51)	6.52 (1.69)	8.82 (2.70)	<0.001^b^*
CRST, rep, $\bar{X}$ (SD)	14.23 (6.40)	9.80 (2.52)	18.67 (6.03)	<0.001^b^*
MBQOA, $\bar{X}$ (SD)	6.62 (5.44)	4.42 (3.37)	8.81 (6.23)	0.001^b^*
ACE-R (0–100), $\bar{X}$ (SD) (range)	68.58 (20.32) (18–97)	51.85 (14.46) (18–81)	85.32 (7.29) (63–97)	<0.001^b^*
GDS (0–15), $\bar{X}$ (SD)	3.02 (2.79)	3.57 (3.18)	2.47 (2.24)	0.082^a^

^a^ Independent-samples t-test; ^b^Mann-Whitney test; ^c^Chi-squared test; *Statistically significant differences (*p* < 0.05).

AD: Alzheimer's disease; BMI: body mass index; 30STS: 30-s sit-to-stand test; CRST: Calf-raise sênior test; MBQOA: Modified Baecke Questionnaire for Older Adults; ACE-R: Addenbrooke's Cognitive Examination - Revised; GDS: Geriatric Depression Scale; $\bar{X}$ : mean; SD: standard deviation; n: number of older adults; kg/m^2^: kilogram per meter square; rep: repetitions.

In the crude models, AD was associated with all knee and ankle isokinetic measurements. The mean isokinetic muscle strength was significantly lower in the with-AD group, given the negative β values (*p* < 0.05). Regarding the R^2^ values, the diagnosis of AD alone was able to explain up to 35.72% of the variation in knee and ankle isokinetic muscle strength. The crude models for each isokinetic muscle strength variable are shown in Table 2.

**Table 2.** Crude models for each isokinetic muscle strength measure.

Isokinetic measures				
Knee at 60°/s (concentric contraction)	β 0 ^a^	β 1 ^a^	*p*	R^2^
Peak Torque (N-m) (Bw), Extension	111.29	−30.75	**<0**.001*	21.31%
Peak Torque (N-m) (Bw), Flexion	54.12	−18.45	**<0**.001*	16.69%
Average Peak Torque, (N-m) (Bw), Extension	100.81	−29.67	**<0**.001*	22.16%
Average Peak Torque (N-m) (Bw), Flexion	49.39	−19.17	**<0**.001*	19.54%
Total Work (J), Extension	310.07	−107.86	**<0**.001*	25.47%
Total Work (J), Flexion	152.11	−70.19	**<0**.001*	20.20%
Knee at 180°/s (concentric contraction)	β 0 ^a^	β 1 ^a^	*p*	R^2^
Peak Torque (N-m) (Bw), Extension	67.96	−21.00	**<0**.001*	23.99%
Peak Torque (N-m) (Bw), Flexion	46.62	−13.81	**0**.001*	13.14%
Average Peak Torque, (N-m) (Bw), Extension	57.14	−18.22	**<0**.001*	23.84%
Average Peak Torque (N-m) (Bw), Flexion	40.02	−14.77	**0**.001*	16.50%
Total Work (J), Extension	530.38	−235.66	**<0**.001*	35.72%
Total Work (J), Flexion	251.11	−155.59	**<0**.001*	20.24%
Knee (isometric contraction)	β 0 ^a^	β 1 ^a^	*p*	R^2^
Peak Torque (N-m) (Bw), Extension	147.55	−26.08	**0**.003*	9.34%
Peak Torque (N-m) (Bw), Flexion	68.70	−8.31	**0**.042*	3.96%
Average Peak Torque, (N-m) (Bw), Extension	141.02	−28.36	**0**.001*	11.24%
Average Peak Torque (N-m) (Bw), Flexion	64.95	−9.67	**0**.016*	6.03%
Ankle at 30°/s (concentric contraction)	β 0 ^a^	β 1 ^a^	*p*	R^2^
Peak Torque (N-m) (Bw), Plantarflexion	66.24	−23.95	**<0**.001*	20.30%
Peak Torque (N-m) (Bw), Dorsiflexion	39.37	−8.26	**0**.001*	13.85%
Average Peak Torque, (N-m) (Bw), Plantarflexion	58.79	−24.42	**<0**.001*	23.32%
Average Peak Torque (N-m) (Bw), Dorsiflexion	35.60	−7.40	**0**.001*	15.93%
Total Work (J), Plantarflexion	92.24	−51.27	**<0**.001*	21.29%
Total Work (J), Dorsiflexion	64.69	−22.74	**<0**.001*	20.47%

^a^ Based on the reference equation of the regression line: y predicted = β**_0_** (intercept) ± β**_1_** (regression coefficient) **·** x + ε (random error); Reference category of the independent variable: older adults without AD; Statistically significant differences (*p* < 0.05).

AD: Alzheimer's disease; N-m: Newton-meter; J: joules; °/s: degree per seconds; Bw: adjusted for body weight.

When the models were adjusted for the control variables selected by univariate analysis (Table 1), AD was associated only with peak torque (β 1 = -16.23; *p* *<* 0.048) and average peak torque (β 1 = -13.65; *p* < 0.049) of the ankle at 30°/s (plantar flexion) in concentric mode. In the model adjusted for peak torque and average peak torque, the clinical control variables handgrip strength and 30STS were also associated, and the older adults with-AD presented a higher mean (positive β, *p* < 0.05). Regarding the R^2^ values, the diagnosis of AD alone was able to explain 48.30% of the variation in peak torque and 51.16% of the variation in the average peak torque of ankle plantar flexors. There was no association between AD and all knee isokinetic measurements at 60°/s and 180°/s (extension and flexion) in concentric and isometric modes, total ankle work at 30°/s (plantar flexion), or with all ankle isokinetic measurements at 30°/s (dorsiflexion) in concentric mode. The adjusted models for each isokinetic muscle strength variable are shown in Table 3. The complete adjusted models for all covariates are presented in Supplemental Material 2.

**Table 3.** Adjusted models for each isokinetic muscle strength measure.

Isokinetic measures				
Knee at 60°/s (concentric contraction)	β 0 ^a^	β 1 ^a^	*p*	R^2^
Peak Torque (N-m) (Bw), Extension	76.11	−14.68	0.234	46.46%
Handgrip strength, kgf		1.43	0.005	
30STS, rep		3.46	0.047	
Peak Torque (N-m) (Bw), Flexion	−14.87	−8.94	0.238	55.55%
Handgrip strength, kgf		1.04	<0.001	
30STS, rep		5.22	<0.001	
Average Peak Torque, (N-m) (Bw), Extension	74.85	−13.38	0.246	47.98%
Handgrip strength, kgf		1.24	0.001	
30STS, rep		4.08	0.013	
Average Peak Torque (N-m) (Bw), Flexion	−7.60	−8.99	0.213	56,69%
Handgrip strength, kgf		0.87	0.001	
30STS, rep		5.05	<0.001	
Total Work (J), Extension	−111.16	−36.43	0.311	56.30%
Handgrip strength, kgf		6.32	<0001	
BMI, kg/m2		4.47	0.04	
Total Work (J), Flexion	−46.43	−37.66	0.187	48.15%
Handgrip strength, kgf		3.47	0.002	
30STS, rep		13.75	<0001	
Knee at 180°/s (concentric contraction)	β 0 ^a^	β 1 ^a^	*p*	R^2^
Peak Torque (N-m) (Bw), Extension	−0.63	−11.23	0.118	56.85%
BMI, kg/m2		−1.29	0.004	
Handgrip strength, kgf		1.27	<0.001	
30STS, rep		2.42	0.017	
Peak Torque (N-m) (Bw), Flexion	1.48	−3.46	0.593	53.02%
BMI, kg/m2		−1.05	0.011	
Handgrip strength, kgf		1.06	<0.001	
30STS, rep		2.49	0.007	
Average Peak Torque, (N-m) (Bw), Extension	−2.57	−8.43	0.174	57.40%
BMI, kg/m2		−1.14	0.004	
Handgrip strength, kgf		0.93	<0.001	
30STS, rep		2.60	0.003	
Average Peak Torque (N-m) (Bw), Flexion	18.41	−4.37	0.478	54.14%
BMI, kg/m2		−0.92	0.018	
Handgrip strength, kgf		0.91	<0.001	
30STS, rep		2.56	0.004	
Total Work (J), Extension	−375.99	−90.26	0.152	61.47%
Handgrip strength, kgf		11.07	<0.001	
30STS, rep		19.18	0.031	
Total Work (J), Flexion	109.29	−57.26	0.355	49.86%
Handgrip strength, kgf		6.82	0.001	
30STS, rep		30.21	0.001	
Knee (isometric contraction)	β 0 ^a^	β 1 ^a^	*p*	R^2^
Peak Torque (N-m) (Bw), Extension	119.73	−11.86	0.447	43.99%
BMI, kg/m2		−2.47	0.013	
Handgrip strength, kgf		2.30	<0.001	
Peak Torque (N-m) (Bw), Flexion	45.42	3.51	0.607	47.54%
Handgrip strength, kgf		1.22	<0.001	
Average Peak Torque, (N-m) (Bw), Extension	86.38	−11.47	0.452	46.52%
BMI, kg/m2		−2.37	0.015	
Handgrip strength, kgf		2.33	<0.001	
Average Peak Torque (N-m) (Bw), Flexion	44.55	3.22	0.627	49.25%
Handgrip strength, kgf		1.17	<0.001	
Ankle at 30°/s (concentric contraction)	β 0 ^a^	β 1 ^a^	*p*	R^2^
Peak Torque (N-m) (Bw), Plantarflexion	93.55	−16.23	**0**.048*	48.30%
Handgrip strength, kgf		1.05	0.001	
30STS, rep		4.94	0.004	
Peak Torque (N-m) (Bw), Dorsiflexion	53.39	−5.67	0.212	33.05%
BMI, kg/m2		−0.87	0.002	
Handgrip strength, kgf		0.36	0.014	
Average Peak Torque, (N-m) (Bw), Plantarflexion	73.56	−13.65	**0**.049*	51.16%
Handgrip strength, kgf		0.86	0.003	
30STS, rep		3.92	0.002	
Average Peak Torque (N-m) (Bw), Dorsiflexion	40.49	−4.23	0.287	27.17%
BMI, kg/m2		−0.54	0.030	
Handgrip strength, kgf		0.28	0.030	
Total Work (J), Plantarflexion	100.98	−31.19	0.158	38.80%
Handgrip strength, kgf		2.49	0.001	
Total Work (J), Dorsiflexion	10.52	−11.65	0.278	28.66%
Handgrip strength, kgf		1.01	0.004	

^a^ Based on the reference equation of the regression line: y predicted = β**_0_** (intercept) ± β**_1_** (regression coefficient) **·** x + ε (random error); Reference category of the independent variable: older adults without AD; Statistically significant differences *(p* *<* 0.05); The models were adjusted for age, BMI, handgrip strength, 30STS, CRST, MBQOA and ACE-R; Only control variables that remained significant in the final adjusted models are presented in Table 3. Additional variables initially included in the models, but that did not reach statistical significance, can be found in Supplemental Material 2.

AD: Alzheimer's disease; BMI: body mass index; 30STS: 30-s sit-to-stand test; CRST: Calf-raise sênior test; MBQOA: Modified Baecke Questionnaire for Older Adults; ACE-R: Addenbrooke's Cognitive Examination - Revised; GDS: Geriatric Depression Scale; N-m: Newton-meter; J: joules; °/s: degree per seconds; Bw: adjusted for body weight; n: number of older adults; kg/m^2^: kilogram per meter square; rep: repetitions.

## Discussion

The main result of this study is that community-dwelling older adults with AD have reduced isokinetic muscle strength of ankle plantar flexors. In older adults without dementia (i.e., healthy), the muscle strength of plantar flexors has a greater impact on postural balance compared to the muscle strength of knee extensors.^6^ After model adjustment, the association between AD and muscle strength remained significant exclusively for the ankle but not for the knee, highlighting the importance of distal muscles in postural control.

Although scarce, there is evidence in the literature that lower ankle dorsiflexion muscle strength is associated with the development of AD over time,^34^ reinforcing the relevance of the findings of the present study. In the literature, a greater number of studies have investigated the association between AD with postural balance,^35^ and with gait,^36, 37^ compared to those that have examined the relationship between AD and muscle strength.

Gait changes in older adults with AD occur due to factors such as increasing age, cognitive decline, neuropsychiatric disorders, etc.^38, 39^ In the literature, there is evidence of an association between muscle weakness and gait alterations in older adults without dementia (i.e., healthy),^40^ but this relationship cannot yet be established as causal in older adults with AD. Therefore, it is suggested that future studies investigate the causal relationship between ankle muscle weakness and gait changes in older adults with AD.

Older adults diagnosed with AD are at an elevated risk of falling compared to cognitively healthy peers.^41^ Existing literature points to factors such as impaired postural control and reduced gait velocity as key contributors to this increased fall risk.^42, 43^ Based on the findings of this study, the observed lower limb muscle weakness in individuals with AD may also play a role in fall susceptibility, underscoring the need for future research to explore this potential link. Moreover, in older adults without cognitive impairment, strength in the ankle plantar flexor muscles has been shown to be protective against falls,^44^ suggesting that this relationship may be relevant to those with AD as well.

This study identified that community-dwelling older adults with AD did not present a reduction in isokinetic muscle strength of knee extensors and flexors. Previous studies have showed that knee extensor and flexor muscle strength is associated with the ability of older adults without dementia (i.e., healthy) to perform activities of daily living (e.g., sitting and standing, going up and down stairs, reaching tasks, etc.).^45–48^ Since no significant difference was observed between groups, we can hypothesize that older adults with AD also possess necessary the levels of muscle strength to perform such tasks. However, more studies are needed to confirm this hypothesis.

On the other hand, Pedroso et al. (2018) observed that older adults with AD exhibit lower limb muscle weakness as assessed by the 30STS clinical functional test, compared to older adults without dementia (i.e., healthy).^49^ However, clinical functional tests do not allow for the separate assessment of knee extensors and flexors as the isokinetic dynamometer does. Thus, the reduction in muscle strength is detected in global assessments but more precise and segmented evaluations using the isokinetic dynamometer do not reveal significant differences This suggests that postural balance may be more affected in AD as captured by the 30STS clinical functional test rather than lower limb muscle strength per se.

In this context, the analysis of the explanatory power of the models contributes to a better understanding of the magnitude of these associations. The R^2^ values in the present study indicated that the AD diagnosis explains a relevant portion of the variability in isokinetic muscle strength, ranging from up to 35.72% in the crude models to 48.30% and 51.16% in the adjusted models for the ankle plantar flexors, reinforcing the importance of the disease in these outcomes. However, the R^2^ values also suggest that other factors, such as cognitive and physical characteristics, may contribute to the variability of lower limb muscle strength, indicating the multifactorial nature of these associations. These findings reinforce the clinical relevance of lower limb muscle weakness in older adults with AD.

With respect to the applicability of these findings, lower limb muscle weakness in older adults with AD warrants particular attention in both scientific research and clinical practice. Disease progression has been associated with impairments in postural balance in this population,^50^ suggesting a potentially similar impact on muscle strength. Accordingly, the implementation of rehabilitation strategies aimed at maintaining muscle strength may yield important functional benefits as has already been demonstrated for postural balance.^51^ Moreover, the fact that this association remained restricted to the ankle muscles after adjustments reinforces the relevance of interventions targeting the distal muscles involved in postural control among older adults with AD.

Finally, we highlight the strengths of this study as the confirmation of the dementia etiology, AD medical diagnosis according to the DSM-IV, AD stage classification according to the CDR, inclusion of the older adults without-AD for comparison purposes, and the use of an adapted and standardized isokinetic assessment protocol for older adults with AD. As limitations of this study, we highlight the absence of evaluator blinding with respect to group allocation (without-AD and with-AD). Knowledge of the AD diagnosis may have introduced expectation bias, particularly in assessments using the isokinetic dynamometer, which depend on maximal voluntary effort, potentially influencing both performance and the conduct of the assessment. This lack of blinding stems from the fact that part of the assessments was carried out in the presence of caregivers of the older adults with AD, thereby making the diagnosis known to the evaluator. Lastly, as with cross-sectional studies, it is not possible to establish causal relationships,^52^ thus, caution is warranted in the interpretation of the associations and their directionality.

### Conclusions

AD is associated with ankle isokinetic muscle strength in community-dwelling older adults. Older adults with AD showed reduced isokinetic muscle strength of ankle plantar flexors. This finding is relevant, given that lower limb muscle weakness may contribute to an increased risk of falls among older adults with AD, although studies specifically assessing ankle muscle strength in this population remain scarce. Therefore, the importance of regular monitoring of ankle muscle strength during physical evaluations, as well as the implementation of interventions to prevent complications, is emphasized. Lastly, no association between AD and isokinetic muscle strength of either ankle dorsiflexors or knee (flexors/extensors) were found in community-dwelling older adults.

## Supplemental Material

sj-docx-1-alz-10.1177_13872877261471888 - Supplemental material for Exploring the relationship between Alzheimer's disease and lower extremity isokinetic muscle strength in community-dwelling older adults: A cross-sectional observational study Supplemental material, sj-docx-1-alz-10.1177_13872877261471888 for Exploring the relationship between Alzheimer's disease and lower extremity isokinetic muscle strength in community-dwelling older adults: A cross-sectional observational study by Marcos Paulo Braz De Oliveira, Silvia Lanziotti Azevedo Da Silva, Natália Oiring De Castro Cezar, Carla Rigo Lima-Truax, Brendon David Truax, Sónia Brito-Costa, Anielle Cristhine De Medeiros Takahashi, Paula Regina Mendes Da Silva Serrão and Larissa Pires De Andrade in Journal of Alzheimer's Disease

## Data Availability

The data used in this study is available upon reasonable request to the corresponding author. Requests will be considered in accordance with ethical, legal, and confidentiality requirements.
