# Supplementary material for: Exploring the relationship between Alzheimer's disease and lower extremity isokinetic muscle strength in community-dwelling older adults: A cross-sectional observational study
Source: J Alzheimers Dis. 2026 Jul 29;113(2):895–905. doi: 10.1177/13872877261471888 (PMC13688339; doi:10.1177/13872877261471888)
Supplement: Supplementary file 1 [file sj-docx-1-alz-10.1177_13872877261471888.docx]

**Supplemental Material**

**Exploring the relationship between Alzheimer’s disease and lower extremity isokinetic muscle strength in community-dwelling older adults: A cross-sectional observational study**

**Supplemental Material 1: Anamnesis Form**

Time:

Date:

Place:

Note:

Name:

Name (responsible):

Adress:

Phone:

Document:

Brazilian Unified Health System (SUS) card:

Date of birth:

Age:

Sex: ( ) female / ( ) male

Years of education:

Ethnicity::

Profession:

Current:

Previous:

Marital status:

Number of children:

Lives alone: ( ) no / ( ) yes

Medications:

( ) does not use

( ) yes, with medical prescription

( ) self-medication

If yes, which ones?

Number of falls: ( ) last 6 months / ( ) last 12 months

Associated diseases:

( ) no

( ) yes, with medical diagnosis

( ) yes, unconfirmed diagnosis

If yes, which ones?

Alcoholic beverage: ( ) never / ( ) former consumer / ( ) former alcoholic / ( ) yes

Smoking: ( ) never / ( ) former smoker / ( ) yes

Injury and/or surgical procedure on the knee and/or ankle? ( ) no / ( ) yes

If yes, which ones?

Osteoporosis: ( ) no / ( ) yes

If yes, do you have bone densitometry?

Multifocal / Bifocal glasses: ( ) no / ( ) yes

Walking aid: ( ) no / ( ) yes

If yes, which ones?

Vertigo: ( ) no / ( ) yes

If yes, how often?

Physical activity: ( ) no / ( ) yes

If yes, how often (times per week / time):

Blood pressure:

Heart rate:

Respiratory rate:

O_2_ saturation:

Weight:

Height:

Body mass index:

Waist size (centimeters):

Hip size (centimeters):

Waist-hip ratio:

| **Supplemental Material 2: Adjusted models for each isokinetic muscle strength measure** | | | | |
| --- | --- | --- | --- | --- |
| Isokinetic measures |  |  |  |  |
| Knee at 60°/s (concentric contraction) | β 0 ^a^ | β 1 ^a^ | *p* | R^2^ |
| Peak Torque (N-m) (Bw), Extension | 76.11 | -14.68 | 0.234 | 46.46% |
| Age, y |  | 0.01 | 0.98 |  |
| BMI, kg/m2 |  | -1.36 | 0.08 |  |
| Handgrip strength, kgf |  | 1.43 | 0.005 |  |
| MBQOA, score |  | 0.75 | 0.21 |  |
| ACE-R, score |  | 0.04 | 0.87 |  |
| GDS, score |  | -0.07 | 0.51 |  |
| 30STS, rep |  | 3.46 | 0.04 |  |
| CRST, rep |  | -0.23 | 0.76 |  |
| Peak Torque (N-m) (Bw), Flexion | -14.87 | -8.94 | 0.238 | 55.55% |
| Age, y |  | 0.14 | 0.65 |  |
| BMI, kg/m2 |  | -0.01 | 0.98 |  |
| Handgrip strength, kgf |  | 1.04 | <0.001 |  |
| MBQOA, score |  | 0.47 | 0.18 |  |
| ACE-R, score |  | -0.10 | 0.51 |  |
| GDS, score |  | 0.25 | 0.69 |  |
| 30STS, rep |  | 5.22 | <0.001 |  |
| CRST, rep |  | -0.62 | 0.20 |  |
| Average Peak Torque, (N-m) (Bw), Extension | 74.85 | -13.38 | 0.246 | 47.98% |
| Age, y |  | -0.16 | 0.73 |  |
| BMI, kg/m2 |  | -1.04 | 0.14 |  |
| Handgrip strength, kgf |  | 1.24 | 0.001 |  |
| MBQOA, score |  | 0.47 | 0.18 |  |
| ACE-R, score |  | 0.03 | 0.87 |  |
| GDS, score |  | -1.19 | 0.23 |  |
| 30STS, rep |  | 4.08 | 0.013 |  |
| CRST, rep |  | -0.31 | 0.67 |  |
| Average Peak Torque (N-m) (Bw), Flexion | -7.60 | -8.99 | 0.213 | 56,69% |
| Age, y |  | 0.04 | 0.87 |  |
| BMI, kg/m2 |  | 0.01 | 0.99 |  |
| Handgrip strength, kgf |  | 0.87 | 0.001 |  |
| MBQOA, score |  | 0.42 | 0.21 |  |
| ACE-R, score |  | -0.08 | 0.58 |  |
| GDS, score |  | -0.25 | 0.68 |  |
| 30STS, rep |  | 5.05 | <0.001 |  |
| CRST, rep |  | -0.56 | 0.22 |  |
| Total Work (J), Extension | -111.16 | -36.43 | 0.311 | 56.30% |
| Age, y |  | 0.35 | 0.82 |  |
| BMI, kg/m2 |  | 4.47 | 0.04 |  |
| Handgrip strength, kgf |  | 6.32 | <0,001 |  |
| MBQOA, score |  | 0.71 | 0.67 |  |
| ACE-R, score |  | 0.41 | 0.58 |  |
| GDS, score |  | -2.29 | 0.46 |  |
| 30STS, rep |  | 9.58 | 0.05 |  |
| CRST, rep |  | -0.96 | 0.67 |  |
| Total Work (J), Flexion | -46.43 | -37.66 | 0.187 | 48.15% |
| Age, y |  | -0.42 | 0.73 |  |
| BMI, kg/m2 |  | 2.90 | 0.10 |  |
| Handgrip strength, kgf |  | 3.47 | 0.002 |  |
| MBQOA, score |  | 1.06 | 0.43 |  |
| ACE-R, score |  | -0.26 | 0.65 |  |
| GDS, score |  | -2.23 | 0.36 |  |
| 30STS, rep |  | 13.75 | <0,001 |  |
| CRST, rep |  | -2.39 | 0.19 |  |
| Knee at 180°/s (concentric contraction) | β 0 ^a^ | β 1 ^a^ | *p* | R^2^ |
| Peak Torque (N-m) (Bw), Extension | -0.63 | -11.23 | 0.118 | 56.85% |
| Age, y |  | 0.54 | 0.08 |  |
| BMI, kg/m2 |  | -1.29 | 0.004 |  |
| Handgrip strength, kgf |  | 1.27 | <0.001 |  |
| MBQOA, score |  | -0.48 | 0.16 |  |
| ACE-R, score |  | 0.15 | 0.31 |  |
| GDS, score |  | 0.44 | 0.46 |  |
| 30STS, rep |  | 2.42 | 0.017 |  |
| CRST, rep |  | -0.05 | 0.89 |  |
| Peak Torque (N-m) (Bw), Flexion | 1.48 | -3.46 | 0.593 | 53.02% |
| Age, y |  | 0.23 | 0.40 |  |
| BMI, kg/m2 |  | -1.05 | 0.011 |  |
| Handgrip strength, kgf |  | 1.06 | <0.001 |  |
| MBQOA, score |  | 0.21 | 0.48 |  |
| ACE-R, score |  | 0.04 | 0.71 |  |
| GDS, score |  | 0.33 | 0.55 |  |
| 30STS, rep |  | 2.49 | 0.007 |  |
| CRST, rep |  | -0.05 | 0.93 |  |
| Average Peak Torque, (N-m) (Bw), Extension | -2.57 | -8.43 | 0.174 | 57.40% |
| Age, y |  | 0.47 | 0.08 |  |
| BMI, kg/m2 |  | -1.14 | 0.004 |  |
| Handgrip strength, kgf |  | 0.93 | <0.001 |  |
| MBQOA, score |  | -0.14 | 0.62 |  |
| ACE-R, score |  | 0.16 | 0.21 |  |
| GDS, score |  | -0.01 | 0.98 |  |
| 30STS, rep |  | 2.60 | 0.003 |  |
| CRST, rep |  | -0.24 | 0.54 |  |
| Average Peak Torque (N-m) (Bw), Flexion | 18.41 | -4.37 | 0.478 | 54.14% |
| Age, y |  | -0.04 | 0.87 |  |
| BMI, kg/m2 |  | -0.92 | 0.018 |  |
| Handgrip strength, kgf |  | 0.91 | <0.001 |  |
| MBQOA, score |  | 0.20 | 0.48 |  |
| ACE-R, score |  | 0.03 | 0.81 |  |
| GDS, score |  | 0.09 | 0.86 |  |
| 30STS, rep |  | 2.56 | 0.004 |  |
| CRST, rep |  | -0.05 | 0.89 |  |
| Total Work (J), Extension | -375.99 | -90.26 | 0.152 | 61.47% |
| Age, y |  | 3.84 | 0.16 |  |
| BMI, kg/m2 |  | -0.10 | 0.97 |  |
| Handgrip strength, kgf |  | 11.07 | <0.001 |  |
| MBQOA, score |  | -1.52 | 0.61 |  |
| ACE-R, score |  | 2.34 | 0.08 |  |
| GDS, score |  | -0.17 | 0.97 |  |
| 30STS, rep |  | 19.18 | 0.031 |  |
| CRST, rep |  | -1.38 | 0.73 |  |
| Total Work (J), Flexion | 109.29 | -57.26 | 0.355 | 49.86% |
| Age, y |  | -2.99 | 0.27 |  |
| BMI, kg/m2 |  | -1.12 | 0.77 |  |
| Handgrip strength, kgf |  | 6.82 | 0.001 |  |
| MBQOA, score |  | 2.03 | 0.49 |  |
| ACE-R, score |  | 0.09 | 0.94 |  |
| GDS, score |  | -4.55 | 0.39 |  |
| 30STS, rep |  | 30.21 | 0.001 |  |
| CRST, rep |  | -3.48 | 0.38 |  |
| Knee (isometric contraction) | β 0 ^a^ | β 1 ^a^ | *p* | R^2^ |
| Peak Torque (N-m) (Bw), Extension | 119.73 | -11.86 | 0.447 | 43.99% |
| Age, y |  | 0,21 | 0,75 |  |
| BMI, kg/m2 |  | -2.47 | 0.013 |  |
| Handgrip strength, kgf |  | 2.30 | <0.001 |  |
| MBQOA, score |  | 0.57 | 0.44 |  |
| ACE-R, score |  | -0.01 | 0.96 |  |
| GDS, score |  | -2.31 | 0.08 |  |
| 30STS, rep |  | 3.63 | 0.09 |  |
| CRST, rep |  | -0.58 | 0.55 |  |
| Peak Torque (N-m) (Bw), Flexion | 45.42 | 3.51 | 0.607 | 47.54% |
| Age, y |  | -0.05 | 0.86 |  |
| BMI, kg/m2 |  | -0.62 | 0.14 |  |
| Handgrip strength, kgf |  | 1.22 | <0.001 |  |
| MBQOA, score |  | 0.62 | 0.06 |  |
| ACE-R, score |  | -0.04 | 0.06 |  |
| GDS, score |  | -0.77 | 0.19 |  |
| 30STS, rep |  | 0.72 | 0.44 |  |
| CRST, rep |  | 0.34 | 0.43 |  |
| Average Peak Torque, (N-m) (Bw), Extension | 86.38 | -11.47 | 0.452 | 46.52% |
| Age, y |  | 0,41 | 0,53 |  |
| BMI, kg/m2 |  | -2.37 | 0.015 |  |
| Handgrip strength, kgf |  | 2.33 | <0.001 |  |
| MBQOA, score |  | 0.54 | 0.45 |  |
| ACE-R, score |  | 0.05 | 0.87 |  |
| GDS, score |  | -2.13 | 0.10 |  |
| 30STS, rep |  | 3.71 | 0.08 |  |
| CRST, rep |  | -0.48 | 0.62 |  |
| Average Peak Torque (N-m) (Bw), Flexion | 44.55 | 3.22 | 0.627 | 49.25% |
| Age, y |  | -0.14 | 0.61 |  |
| BMI, kg/m2 |  | -0.55 | 0.18 |  |
| Handgrip strength, kgf |  | 1.17 | <0.001 |  |
| MBQOA, score |  | 0.54 | 0.08 |  |
| ACE-R, score |  | -0.01 | 0.99 |  |
| GDS, score |  | -0.79 | 0.17 |  |
| 30STS, rep |  | 0.98 | 0.29 |  |
| CRST, rep |  | 0.22 | 0.60 |  |
| Ankle at 30°/s (concentric contraction) | β 0 ^a^ | β 1 ^a^ | *p* | R^2^ |
| Peak Torque (N-m) (Bw), Plantarflexion | 93.55 | -16.23 | **0.048*** | 48.30% |
| Age, y |  | -0.61 | 0.14 |  |
| BMI, kg/m2 |  | -0.81 | 0.18 |  |
| Handgrip strength, kgf |  | 1.05 | 0.001 |  |
| MBQOA, score |  | 0.12 | 0.79 |  |
| ACE-R, score |  | -0.27 | 0.18 |  |
| GDS, score |  | -0.36 | 0.66 |  |
| 30STS, rep |  | 4.94 | 0.004 |  |
| CRST, rep |  | 0.12 | 0.84 |  |
| Peak Torque (N-m) (Bw), Dorsiflexion | 53.39 | -5.67 | 0.212 | 33.05% |
| Age, y |  | -0.08 | 0.66 |  |
| BMI, kg/m2 |  | -0.87 | 0.002 |  |
| Handgrip strength, kgf |  | 0.36 | 0.014 |  |
| MBQOA, score |  | 0.01 | 0.93 |  |
| ACE-R, score |  | -0.01 | 0.85 |  |
| GDS, score |  | -0.02 | 0.94 |  |
| 30STS, rep |  | 0.80 | 0.20 |  |
| CRST, rep |  | 0.07 | 0.79 |  |
| Average Peak Torque, (N-m) (Bw), Plantarflexion | 73.56 | -13.65 | **0.049*** | 51.16% |
| Age, y |  | -0.58 | 0.13 |  |
| BMI, kg/m2 |  | -0.52 | 0.35 |  |
| Handgrip strength, kgf |  | 0.86 | 0.003 |  |
| MBQOA, score |  | 0.40 | 0.34 |  |
| ACE-R, score |  | -0.19 | 0.30 |  |
| GDS, score |  | -0.57 | 0.45 |  |
| 30STS, rep |  | 3.92 | 0.002 |  |
| CRST, rep |  | 0.06 | 0.91 |  |
| Average Peak Torque (N-m) (Bw), Dorsiflexion | 40.49 | -4.23 | 0.287 | 27.17% |
| Age, y |  | -0.06 | 0.70 |  |
| BMI, kg/m2 |  | -0.54 | 0.030 |  |
| Handgrip strength, kgf |  | 0.28 | 0.030 |  |
| MBQOA, score |  | 0.02 | 0.89 |  |
| ACE-R, score |  | 0.02 | 0.78 |  |
| GDS, score |  | -0.01 | 0.97 |  |
| 30STS, rep |  | 0.56 | 0.30 |  |
| CRST, rep |  | 0.03 | 0.89 |  |
| Total Work (J), Plantarflexion | 100.98 | -31.19 | 0.158 | 38.80% |
| Age, y |  | -1.20 | 0.21 |  |
| BMI, kg/m2 |  | 0.02 | 0.98 |  |
| Handgrip strength, kgf |  | 2.49 | 0.001 |  |
| MBQOA, score |  | -0.19 | 0.85 |  |
| ACE-R, score |  | -0.26 | 0.57 |  |
| GDS, score |  | -1.52 | 0.42 |  |
| 30STS, rep |  | 4.79 | 0.12 |  |
| CRST, rep |  | 0.04 | 0.97 |  |
| Total Work (J), Dorsiflexion | 10.52 | -11.65 | 0.278 | 28.66% |
| Age, y |  | -0.13 | 0.76 |  |
| BMI, kg/m2 |  | 0.80 | 0.23 |  |
| Handgrip strength, kgf |  | 1.01 | 0.004 |  |
| MBQOA |  | -0.23 | 0.64 |  |
| MBQOA, score |  | 0.15 | 0.49 |  |
| ACE-R, score |  | 0.25 | 0.78 |  |
| GDS, score |  | 0.68 | 0.64 |  |
| CRST, rep |  | -0.16 | 0.80 |  |
| ^a^Based on the reference equation of the regression line: y predicted = β₀ (intercept) ± β₁ (regression coefficient) · x + ε (random error); Reference category of the independent variable: older adults without AD; *Statistically significant differences (*p* < 0.05); The models were adjusted for age, BMI, handgrip strength, 30STS, CRST, MBQOA, and ACE-R.  AD: Alzheimer’s disease; BMI: body mass index; 30STS: 30-seconds sit-to-stand test; CRST: Calf-raise sênior test; MBQOA: Modified Baecke Questionnaire for Older Adults; ACE-R: Addenbrooke’s Cognitive Examination - Revised; GDS: Geriatric Depression Scale; N-m: Newton-metre; J: joules; °/s: degree per seconds; Bw: adjusted for body weight; n: number of older adults; kg/m^2^: kilogram per meter square; (rep) repetitions | | | | |
